# Phenology of brown marmorated stink bug described using female reproductive development

**DOI:** 10.1002/ece3.3125

**Published:** 2017-07-21

**Authors:** Anne L. Nielsen, Shelby Fleischer, George C. Hamilton, Tori Hancock, Gregorz Krawczyk, Jana C. Lee, Emily Ogburn, John M. Pote, Amy Raudenbush, Ann Rucker, Michael Saunders, Victoria P. Skillman, Jeanne Sullivan, Jody Timer, James Walgenbach, Nik G. Wiman, Tracy C. Leskey

**Affiliations:** ^1^ Department of Entomology Rutgers University New Brunswick NJ USA; ^2^ Department of Entomology Pennsylvania State University University Park PA USA; ^3^ USDA ARS Appalachian Fruit Research Station Kearneysville WV USA; ^4^ USDA ARS Horticultural Crops Research Unit Corvallis OR USA; ^5^ Mountain Horticultural Crops Research and Extension Center North Carolina State University Mills River NC USA; ^6^ Ohio State University Wooster OH USA; ^7^ Oregon State University Corvallis OR USA; ^8^ West Virginia Weselyan College Buckhannon WV USA

**Keywords:** biofix, brown marmorated stink bug, degree‐day model, invasive, Pentatomidae, physiology

## Abstract

Temperature‐based degree‐day models describe insect seasonality and to predict key phenological events. We expand on the use of a temperature‐based process defining timing of reproduction through the incorporation of female reproductive physiology for the invasive pentatomid species *Halyomorpha halys,* the brown marmorated stink bug. A five‐stage ranking system based on ovary development was able to distinguish between the reproductive statuses of field‐collected females. Application of this ranking method described aspects of *H. halys*’ seasonality, overwintering biology, and phenology across geographic locations. Female *H. halys* were collected in the US from NJ, WV, NC, OR, and two sites in PA in 2006–2008 (Allentown, PA only) and 2012–2014. Results identify that *H. halys* enters reproductive diapause in temperate locations in the fall and that a delay occurs in developmental maturity after diapause termination in the spring. Modification of the Snyder method to identify biofix determined 12.7‐hr photoperiod as the best fit to define initiation of reproduction in the spring. Applying the biofix, we demonstrated significant differences between locations for the rate at which the overwintering generation transition into reproductive status and the factors contributing to this difference require further study. For example, after including abiotic variables influencing development such as temperature and photoperiod (critical diapause cue), reproduction occurred earlier in OR and for an extended period in NJ. This data describe a method to investigate insect seasonality by incorporating physiological development across multiple regions that can clarify phenology for insects with overlapping generations.

## 1 INTRODUCTION

Insect activity as measured by dispersal, foraging, feeding, and development is dependent on abiotic conditions, specifically temperature and photoperiod/day length. Together these predictable seasonal activity patterns can be described as phenology (Wolda, 1988). Based on the relationship between available food resources, and the abiotic factors of temperature and photoperiod, the phenology of a particular species distributed across a large geographic area can vary. The description of phenology across such scales is especially important for understanding species biology as individuals adapt and interact within a novel or changing habitat. For invasive species, phenology may be strongly influenced not only by its current interactions with the invaded environment but also the specific phenotype expressed by the invasive population. If a genetic bottleneck occurs, the specific phenotype(s) of the introduced population may determine characteristics that regulate successful adaptation or maladaptation in the novel environment as has been shown for a limited number of invasive species (Lee, 2002; Musolin & Saulich, 2012). In agriculture, the measurement of these processes (development and seasonality as determined by phenotype) plays an important role in the development and implementation of pest management programs designed to target a specific life stage to prevent damage, limit population growth, or both.

As exothermic organisms, insect development is dependent upon temperature which can be recorded as growing degree‐days calculated based on the daily accumulated heat units received above a physiologically meaningful lower threshold (developmental temperature minimum [*T*_o_]) by the insect. Degree‐day (DD) models require the known thermal units (i.e., *T*_o_ and developmental temperature maximum [*T*_m_]) for development as well as a biofix, or the point at which DD accumulation or development begins. Insects with long duration of life stages and/or long survival times may have strongly overlapping generations which make estimations of phenology and application of DD models for a population difficult. The incorporation of female reproductive state into studies of insect phenology has clarified critical phenological periods for many species, including rice hoppers (*Sogatella furcifera* (Horvath) (Hemiptera: Membracidae) and *Nilaparvata lugens* (Stål) (Hemiptera: Membracidae)) (Zheng et al., 2014) and *Euschistus conspersus* (Uhler) (Hemiptera: Pentatomidae) (Cullen & Zalom, 2006). This approach may be a useful tool for defining phenology across geographic locations of *Halyomorpha halys* (Stål) (Hemiptera: Pentatomidae), an invasive species accidentally introduced to North America and Europe (Gariepy, Fraser, & Scott‐Dupree, 2014; Hoebeke & Carter, 2003; Maistrello, Dioli, Bariselli, Mazzoli, & Giacalone‐Forini, 2016).

The first detections of *H. halys* in the eastern United States occurred in the late 1990s, and it has since become widespread throughout the United States (Hoebeke & Carter, 2003; Leskey, Short, Butler, & Wright, 2012). Populations in the mid‐Atlantic region increased to damaging levels beginning in 2008 with a population outbreak in 2010 that caused significant losses to fruit and vegetable crops, costing nearly $37 million USD in apple alone (Association, 2011; Leskey, Hamilton, et al., 2012; Nielsen & Hamilton, 2009b; Rice et al., 2014). Since 2010, populations in the mid‐Atlantic region have declined, which may be due to a response from natural enemies or the implementation of intense management programs (Leskey, Hamilton, et al., 2012; Ogburn et al., 2016). Outside of the mid‐Atlantic, populations in the Pacific Northwest (specifically Oregon) and the Southeast increased after 2010 to economically important levels. As of 2008, the population affecting the eastern US is purported to have originated from a single introduction of a small propagule size, whereas the West Coast US population is from separate multiple introductions and is comprised of different haplotypes (Valentin, Nielsen, Wiman, Lee, & Fonseca, *in review*; Xu, Fonseca, Hamilton, Hoelmer, & Nielsen, 2014). Limited haplotype heterogeneity could provide an adaptive advantage or a genetic bottleneck for the species. As a long‐lived species with overlapping generations (Haye, Abdallah, Gariepy, & Wyniger, 2014; Nielsen & Hamilton, 2009a), field‐based comparative information on *H. halys* phenology across its invaded habitat in the US is unavailable. A stage‐specific population dynamics model developed for *H. halys* was based primarily on the developmental, fecundity, and survivorship parameters. The model predicts the capacity for bivoltinism, with the interaction of diapause cues and temperature causing population growth to vary significantly among the eight US locations modeled (Nielsen, Fleischer, & Chen, 2016). Model predictions, however, were sensitive to functions estimating termination and induction of diapause, and the rate at which the population transitions between a diapausing and reproductive state was assumed to be constant across the geographic range.

The objective of this research is to use female reproductive seasonality in the field to understand key phenological periods for *H. halys* and to determine whether phenological differences occur between geographic locations in North America. We validate a revised female reproductive ranking system and (1) identify reproductive status of field‐collected females entering and terminating diapause, (2) identify a population biofix for initiation of reproductive development that can be used in forecasting models, and (3) identify the voltinism of *H. halys* populations at multiple geographic locations.

## 2 METHODS

### 2.1 Specimen collection

Collection of females occurred in 2006–2008 in Allentown, PA and in 2012–2014 at multiple locations. Female *H. halys* collected from building interiors or exteriors (overwintering habitat), and plant material at the Rodale Organic Tree Center in Allentown, PA (“PAA”), Rutgers Agricultural Research and Extension Center in Bridgeton, NJ (“NJ”); USDA Appalachian Fruit Research Center in Kearneysville, WV (“WV”); Penn State Fruit Research and Extension Center in Biglerville, PA (“PAB”); in and around the Mountain Research Station in Asheville, NC (“NC”) and the Willamette Valley, Oregon (“OR”) were assessed (Table 1). A total of 5,911 female *H. halys* were collected from NC, PAA, PAB, NJ, WV, and OR over multiple years (Table 1). Of these, reproductive rankings were classified for 5,682 and weight recorded for 5,716 females.

**Table 1:** Collection site and number of female *Halyomorpha halys* used for dissections

Abbreviation	Location	Coordinates	Year	*n*
NC	Asheville, NC	35.42°N 82.56°W	2013	315
			2014	240
NJ	Bridgeton, NJ	39.52°N 75.20°W	2012	607
			2013	952
			2014	190
OR	Willamette Valley (Aurora, OR)	45.23°N 122.75°W	2012	24
			2013	85
			2014	87
PAA	Allentown, PA	40.55°N 75.52°W	2006	61
			2007	264
			2008	552
			2012	534
			2013	718
PAB	Biglerville, PA	39.93°N 77.25°W	2013	544
WV	Kearneysville, WV	39.35°N 77.88°W	2012	471
			2013	205
			2014	62
Total				5,911

Females were collected by hand, canvas beating sheet (71 cm × 71 cm, BioQuip, Rancho Dominguez, CA), 25 watt compact fluorescent light traps in full spectrum white, ultra violet black or visible blue or 1.2 m high black pyramid traps baited with experimental aggregation pheromone for *H. halys* (“pheromone trap”). Collection times and frequency varied but were conducted beginning in April through the end of October. Collection source (host plant or overwintering habitat) was not recorded for all specimens or years. We recorded body mass (g) of live females within a few hours of collection. In 2006 and 2007, specimens were stored in 70% ethanol, which desiccated some tissues. Beginning in 2008, specimens were killed and immediately dissected or frozen for later dissection.

### 2.2 Female reproductive status

**Figure 1:**
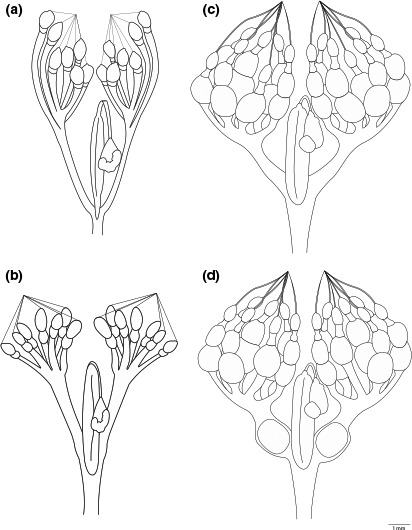
Schematic of *Halyomorpha halys* female reproductive system: (a) Rank 1 with one immature oocyte per ovariole; (b) Rank 2 with more than one immature oocyte per ovariole; (c) Rank 3 with at least one mature oocyte per ovariole; (d) Rank 4 with mature oocytes and at least one oocyte in the lateral oviducts

Females were ranked into five categories of reproductive status with 1 and 2 being previtellogenic, 3 and 4 as vitellogenic or reproductive, and 5 as postreproductive. The ranking system was developed through modification of pentatomid ovarian rankings described by Katayama, Fukuda, and Nozawa (1993), Cullen and Zalom (2006), and Esquivel (2009) and by initial dissection and classification of *H. halys* females of known ages from colony‐reared adults into five reproductive ranks, most similar to Kiritani (1963). Rank 1 females contained a maximum of one immature oocyte per ovariole, the ovaries appeared very undeveloped with lateral and common oviducts thin and usually translucent, and spermatheca appeared very thin (Figure 1a). Rank 2 females contained more than one immature oocyte per ovariole (Figure 1b), the lateral and common oviducts were thicker, but no mature oocytes were present, which was determined by using forceps to determine whether oocyte was hardened. Females in ranks 1 and 2 were classified as previtellogenic. Rank 3 females had at least one mature oocyte per ovariole, the lateral and common oviducts were thicker and not translucent (Figure 1c). If the corpus luteum was present, a female is a minimum of rank 3 as she is vitellogenic. Rank 4 females contained mature oocytes and at least one oocyte in the lateral oviducts and the ovaries contained many eggs (Figure 1d). Females in ranks 3 and 4 were vitellogenic, and rank 5 was postvitellogenic. Rank 5 females had distended ovaries and oviducts, and there was an inconsistent number of oocytes; these females are postvitellogenic. In ranks 1–4, the development of oocytes is symmetrical between ovaries. Diagrams of ranks 1–4 (Figure 1) were drawn from digital images taken with a Cannon D60 camera attached to a Leica MZ8 stereo dissecting scope. Rank 5 was too variable between specimens to definitively characterize with an illustration (photographic examples in Figure S1).

Spermatheca width was used to determine mated status which was categorized visually as unmated (0), possibly mated (1), and mated (2). Females that were not suspected to have mated had a spermatheca that was not distended and was still translucent. Females that were suspected to have mated had an enlarged spermatheca that was opaque. Females that had possibly mated had a slightly distended or somewhat opaque spermatheca.

Insect legs and wings were removed prior to dissection and the insect was pinned ventral side‐down in a Petri dish. Ringer's solution (1 L distilled water, NaCl 9.1 g/L, KCl 0.52 g/L, CaCl_2_ 0.2 g/L, MgCl_2_ 0.8 g/L) was used for all dissections and measurements. We used a stereo dissecting microscope at fixed magnifications with an ocular or stage micrometer for measurements on the maximum pronotum and spermatheca width. Preliminary evaluations demonstrated that spermatheca length did not change over time (ALN unpublished).

Female reproductive development as described by reproductive rank, mated status, and presence of corpus luteum were categorized by accumulated sine degree‐day. Daily maximum and minimum air temperature data for each location/year were collected either directly from research centers or downloaded from NOAA (Table 1). Degree‐days were calculated according to the sine‐wave function (Baskerville & Emin, 1969) with *T*_o_ = 14.17°C as described by Nielsen, Hamilton, and Matadha (2008).

### 2.3 Biofix estimation

The estimation method for estimating a photoperiod biofix was a modification of the standard deviation in degree‐days (*SD*_DD_) method which suggests that the optimal minimum developmental threshold for an insect is that which minimizes the standard deviation across instances in the DD requirement for a phenological event (Snyder, Spano, Cesaraccio, & Duce, 1999). This method was adjusted to find the optimal biofix, rather than minimum developmental threshold, and the calculation was modified to include a scalar term to account for variation in sample size across instances. We modeled a range of biofix estimates from January 1 (no influence of photoperiod) and photoperiods at 0.1‐hr increments from 12.0–14.5 h. Scaling the data based on sampling effort increases the statistical weight of instances with high sample sizes prior to critical vitellogenesis, which should have more accurate estimates of the true date of critical vitellogenesis (Table S1). Modified *SD*_DD_ was calculated using the following equation:

$$\text{Std}\;{\text{Dev}}_{j}=\sqrt{\frac{1}{n}\sum_{i=1}^{n}p_{i}{\left(x_{ij}-{\overset{¯}{x}}_{j}\right)}^{2}}$$

where *i* is the instance, a numerical identifier for each unique combination of location and year, *n* is the total number of instances, *x*_*ij*_ is the DD accumulation on the date of critical vitellogenesis for instance *i* with biofix photoperiod of *j,* ${\overset{¯}{x}}_{j}$ is the mean DD accumulation for critical vitellogenesis with biofix photoperiod *j*, and *p*_*i*_ is the proportion of all *H. halys* females sampled prior to and including the day of critical vitellogenesis made up by the samples of instance *i* (Table 2).

**Table 2:** Mated status of females based on spermatheca category by collection source. Categories are 0—unmated, 1—indeterminate, 2—mated. Mated females are categories 1 and 2 combined. Mated status of females was significantly influenced by collection source

Source	Assigned category of mating status			Mated	
	0	1	2		
Host plant	2,171	113	950	1,063	*n*
	43.24	2.25	18.92	21.17	Total %
	67.13	3.49	29.38	32.87	Row %
Light trap	18	3	29	32	*n*
	0.36	0.06	0.58	0.64	Total %
	36	6	58	64	Row %
Overwintering habitat	1,319	10	14	24	*n*
	26.27	0.2	0.28	0.48	Total %
	98.21	0.74	1.04	1.79	Row %
Pheromone trap	283	14	97	111	*n*
	5.64	0.28	0.193	0.473	Total %
	71.83	3.55	24.62	28.17	Row %
Total	3,791	140	1,090	1,230	*n*
	75.5	2.79	21.71	24.5	Total %

### 2.4 Data analysis

Measurements of body mass and spermatheca width was analyzed with a one‐way ANOVA and means separation with Tukey's HSD. Where data did not meet assumptions of normality, Kruskal–Wallis analysis was performed. Pearson's correlation analysis was performed on the relationship between collection source (host plant, overwintering habitat, light trap, pheromone trap) and mated status or reproductive rank. Data were analyzed in JMP Pro 11 (SASInstitute, 2008).

Summary statistics including the DD calculations and application of the modified Snyder methods were performed in R (Pote, Nielsen, & Grieshop, 2016; Snyder et al., 1999). Comparison of phenology of the overwintering generation across locations was analyzed with logistic regression of vitellogenic females by DD (rounded to 50 DD) up to 650 DD_14.17_ using GLM model with a binomial distribution in R. Only specimens from 2012–2014 were included for sites where collections occurred over multiple years. Model effects with site and year parameters were included and the model producing the lowest AIC value was selected. Tukey's HSD for means separation was used to determine location‐specific differences in the probability of the rate of vitellogenesis phenology.

## 3 RESULTS

A total of 5,911 female *H. halys* were collected from NC, PAA, PAB, NJ, WV, and OR over multiple years (Table 1). Of these, reproductive rankings were classified for 5,678 females and body mass recorded for 5,845 females.

### 3.1 Physiological characteristics

**Figure 2:**
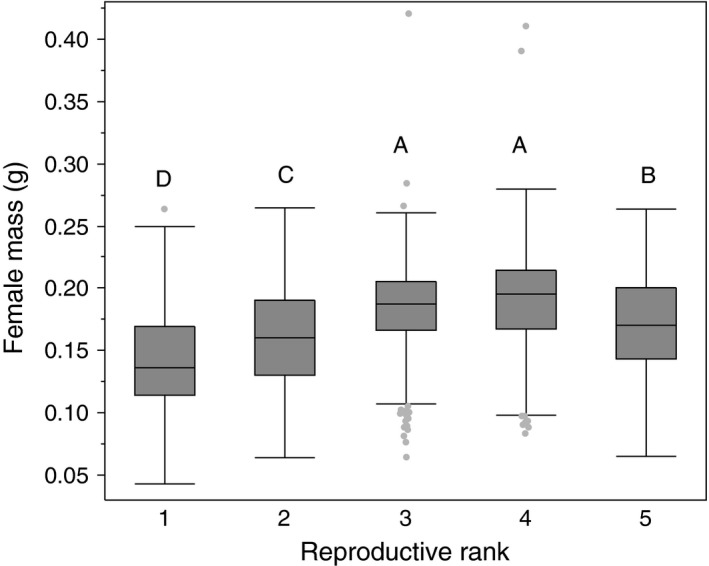
Mean (±*SEM*) live female *Halyomorpha halys* body mass and assigned reproductive ranks from field‐collected specimens from 2006–2008, 2012–2014. Mean values superscribed by the same letters are not significantly different ANOVA, Tukey's HSD *p < *.05)

There was a significant relationship between female body mass and reproductive rank (*F *=* *299.05, *df* = 4, 5162, *p *<* *.0001). Females collected from light traps and pheromone traps were excluded from this analysis as they typically died prior to collection and were desiccated which impacts body mass measurements. All categories differed significantly from each other for body mass except for rank 3 and rank 4 (Figure 2).

**Figure 3:**
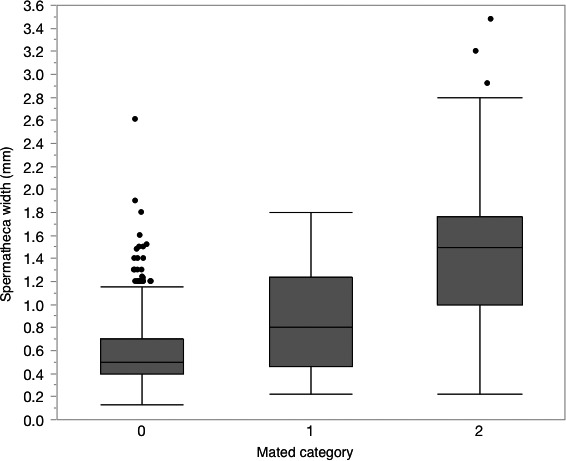
Box plot of spermatheca width assigned to mated category of *Halyomoprha halys* females collected from 2006–2008, 2012–2014. Mean values were significantly different, ANOVA *p *<* *.05

Spermatheca width was measured from 4,256 individuals. There was a significant relationship between the assigned mated status and spermatheca width analysis of mated status (*F *=* *2267.22, *df* = 2, 4255, *p *<* *.0001). Each mated status category was significantly different from the other with the mean spermatheca width (±*SEM*) of unmated females measuring 0.542 (±0.003) mm and the mean width of mated females as 1.371 (±0.018) mm (Figure 3).

Collected females were assigned mated status and reproductive rank by their collection source (i.e., host plant, light trap, overwintering habitat, or pheromone trap). The total number of females assigned to the morphological characteristics may differ between mated status and reproductive rank because in a few cases, the quality of the specimens precluded reproductive rank from being determined. Mated status of females (*n *=* *5,021) was significantly correlated to collection source (*X* ^2^ = 545.03, *df* = 6, *p < *.0001). The majority of collected females were unmated (75.50%) with 21.72% having an enlarged spermatheca indicative of mating (Table 2). Females caught in light traps (*n *=* *50), pheromone traps (*n *=* *394), and on host plants (*n *=* *3,234) were mated (categories 1 and 2 combined) 64.0, 28.17 and 32.89% of the time, respectively. Only 1.79% of females collected in overwintering habitat (*n *=* *1,343) were mated. Within overwintering habitat, the majority of females (98.21%) were unmated, indicating that females enter and terminate diapause states unmated.

The collection source was recorded for a large number of females as host plant (*n *=* *3,234), overwintering habitat (*n *=* *1,343), light trap (*n *=* *44), or pyramid pheromone trap (*n *=* *364) (Table 3). There was a significant relationship between collection source (host plant, overwintering habitat, light trap, or pheromone trap) and reproductive rank (*X* ^2^ = 473.21, *df* = 12, *p < *.0001). Of the 4,983 females that recorded collection source, 64.86% of females were collected from host plants (Table 3). The majority of these (65.07%) were in rank 1, while 24.47% were reproductive (ranks 3 and 4). Females collected from light traps or pheromone traps comprised a low proportion of the samples. Female reproductive rank in pheromone traps reflected host plant collections suggesting that responders to the pheromone are representative of the population that is actively foraging. Females collected in overwintering habitats were predominantly previtellogenic with 99.70% of overwintering females being in ranks 1 and 2. This was true for females collected in overwintering habitats in both the fall and again in the spring as the insects were entering and leaving overwintering habitats, respectively. Only four vitellogenic females (0.09%) were collected from overwintering habitats; however, the collection dates (between 29 May and 4 June 2013) indicate these females were likely resting on a building at a time when most adults were foraging within the environment and should not be considered to have been collected in overwintering structures.

**Table 3:** Reproductive rank of females by collection source. Female reproductive ranks were classified as 1—ovarioles containing one immature oocyte per ovariole; 2—ovarioles containing more than one immature oocyte per ovariole; 3—ovarioles containing at least one mature oocyte per ovariole; 4—ovarioles containing mature oocytes and at least one oocyte in the lateral oviducts; 5—mature female with distended ovaries, corpus luteum present, unequal oocyte number. There was a significant influence of collection source on reproductive

Collection source	Reproductive rank					
	1	2	3	4	5	
Host plant	2,103	268	500	291	70	*n*
	42.20	5.38	10.03	5.84	1.40	Total %
	65.07	8.29	15.47	9.00	2.17	Row %
Light trap	17	11	14	2	0	*n*
	0.34	0.22	0.28	0.04	0	Total %
	38.64	25	31.82	4.54	0	Row %
Overwintering habitat	1,177	162	4	0	0	*n*
	23.62	3.25	0.08	0	0	Total %
	87.64	12.06	0.30	0	0	Row %
Pheromone trap	244	33	54	19	14	*n*
	4.90	0.66	1.08	0.38	0.28	Total %
	67.03	9.07	14.84	5.22	3.85	Row %
Total	3,541	474	572	312	84	*n*
	71.06	9.51	11.48	6.26	1.69	Total %

### 3.2 Estimation of biofix

The lowest modified standard deviation (*SD*_DD_ method) was obtained from calculations assuming a biofix photoperiod of 12.7 h as the best fit for the data (Table S2). This estimated biofix was used in subsequent DD accumulation estimates.

**Figure 4:**
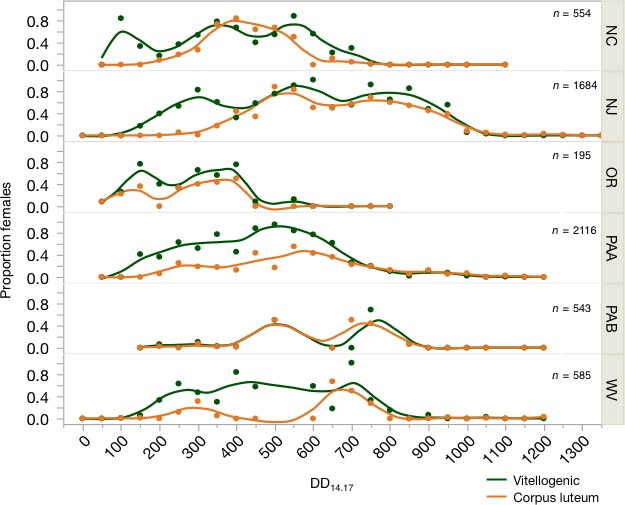
Mean proportion number of vitellogenic female *Halyomorpha halys* across geographic locations by accumulated DD_14.17_ with biofix of 12.7‐hr photoperiod. Green line indicates vitellogenic (ranks 3 and 4) females and orange line indicates the presence of the corpus luteum

**Figure 5:**
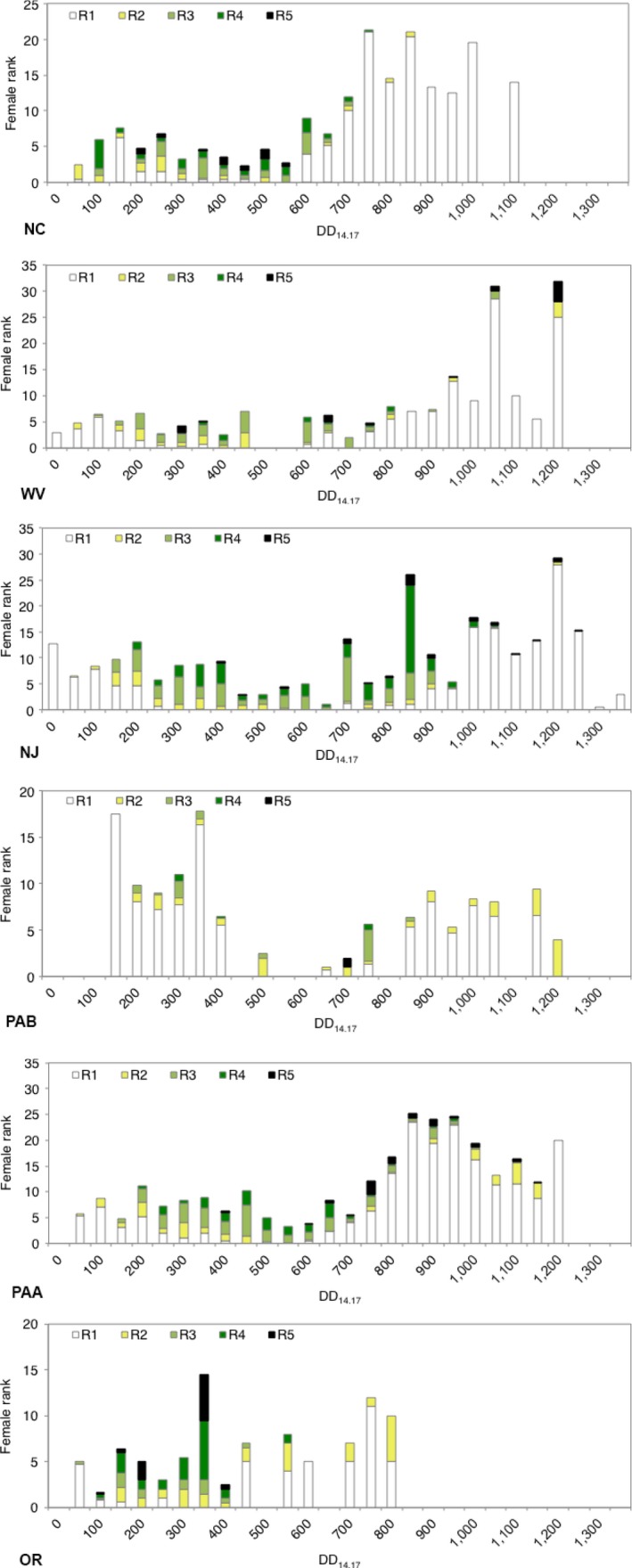
Mean number of female *Halyomorpha halys* in each reproductive rank category by accumulated DD_14.17_ for each location. White bars indicate rank 1, yellow—rank 2, light green—rank 3, dark green—rank 4, black—rank 5

The proportion of vitellogenic females (rank 3 plus 4) and females that had previously oviposited, as indicated by the presence of the corpus luteum, and reproductive rank in relation to DD accumulation was calculated using a 12.7‐hr photoperiod as the biofix for each location (Figures 4 and 5, Table S2). The mean proportion of vitellogenic females at each location varied over time, but initiated approximately at 200 DD_14.17_ except OR and NC which started at 150 DD and PAB which started around 450 DD_14.17_ at the end of June (Figures 4 and 5). Differences observed in phenology in PAB may be an effect of sampling. Describing this dataset by reproductive rank demonstrates that females are entering and exiting diapause in a reproductively immature state (ranks 1 and 2) (Figure 5). We were unable to clearly distinguish between generations through this method, suggesting strongly overlapping generations in the adult stage. However, a rise or peak in rank 1 females later in the season suggests the emergence of the summer adults. Rank 1 females were present at 600–700 DD_14.17_ at most sites, except Oregon which had rank 1 females present beginning at 400 DD_14.17_.

**Figure 6:**
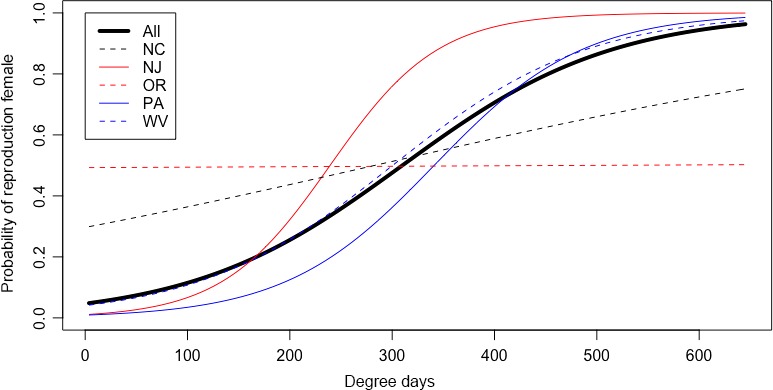
Predicted vitellogenic (ranks 3–5) female *Halyomorpha halys* for the overwintering generation, by geographic location from logistic regression

Reproductive phenology (rate of progression into ranks 3–5) of the overwintering generation (those emerging from diapause up to 650 DD_14.17_) varied significantly by location (AIC = 1,503; year: *df* = 2, *p *=* *.023; DD: *df* = 1, *p *<* *.001; year: *df* = 2, *p *<* *.001; site: *df* = 4, *p *<* *.001; DD × site: *df* = 4, *p *<* *.001) (Figure 6). We expect that DD would change significantly with time as reproductive maturity increases over time. However, the rates at which this occurs were different than hypothesized. OR and NC were not significantly different from each other and represent the most western and southern sites, respectively. NJ, PA, and WV were all significantly different than OR and NC but were not significantly different in the rates of vitellogenesis; however, PA and WV were significantly different from each other at *p *=* *.01. These results may be a reflection of sampling effort, evidence that biotic conditions such as suitable host plants influence early season population dynamics, or localized adaptation to abiotic conditions.

## 4 DISCUSSION

The results of this study validate the female ranking system and provide a descriptive account of *H. halys* phenology based from female reproduction. Although similar to other ranking systems that have been developed for Pentatomidae (Katayama et al., 1993; Kiritani, 1963; Toscano & Stern, 1980), our method was able to statistically distinguish between reproductive ranks by body mass. Swelling of the spermatheca, which became increasingly apparent at rank 2, easily identified mated females indicating that females may mate before becoming reproductively mature. Unlike previous reports, the corpus luteum or “black spot” was not as apparent in *H. halys* as in *Nezara viridula* (L.) (Esquivel, 2009; Kiritani, 1963) or *E. conspersus* (Cullen & Zalom, 2006; Toscano & Stern, 1980), which confounds differentiation of generations.

The life history characteristics of an organism play a significant role in understanding the seasonal patterns and their distributions (Danks, 2007; Wolda, 1988). Diapause induction and termination cues, especially for species that overwinter in the adult stage, are likely extrinsic factors such as temperature and photoperiod (Tauber & Tauber, 1976). Our data support that finding that *H. halys* uses photoperiod as a diapause termination cue as adults emerge from overwintering in the US short‐day photoperiod as the diapause initiation cue has been identified in many species (reviewed in Koštál (2011) and Danks (2007)) including Pentatomidae (Musolin & Saulich, 2012). It is suspected that photoperiod is also a critical cue for diapause termination for *H. halys* but this study did not investigate that..

Our results demonstrate that in the US latitudes studied here, overwintering *H. halys* adults are present and active in the field while retaining a diapausing physiological state well into the spring (April through May) before the initiation of reproduction. Mating and reproduction are indications of diapause termination and postdiapause development (Musolin & Saulich, 2012), and our results conservatively estimate that reproductive development for *H. halys* is triggered by a photoperiod cue of 12.7 hr when *H. halys* is in facultative diapause. Previous estimates have ranged from 13.5‐hr to 14.75‐hr photoperiod within the native range (Niva & Takeda, 2003; Watanabe, 1979; Yanagi & Hagihara, 1980) and the shorter photoperiod we identified may be due to the 10% threshold for vitellogenesis. This estimate is supported by laboratory data on photoperiodic cues for diapause termination in *H. halys* (Nielsen and Walgenbach unpub). After diapause is terminated, reproductive development is primarily driven by temperature, although nutrition (i.e., host plant quality) plays a significant role in development and survivorship of *H. halys* nymphs (Acebes‐Doria, Leskey, & Bergh, 2016) and thus may be critical for post diapause reproductive development as well (Numata, 2004). With an invasive species that has undergone a significant genetic bottleneck, as occurred with the Eastern population of *H. halys* (Xu et al., 2014), there may be increased selection pressure for adaptive phenological traits, such as postdiapause development (Bradshaw & Holzapfel, 2001; Lee, 2002; Urbanski et al., 2012) and we identified differences in the rate of vitellogenesis between geographic regions.

Once reproductive, female *H. halys* continue to reproduce for an estimated 4 months postdiapause termination (Haye et al., 2014; Nielsen et al., 2008). Indeed, vitellogenic females were found for multiple months across locations and years, but the initiation and cessation of reproduction differed between sites. There was, however, a noticeable increase in reproductive rank 1 females across locations at about 600–700 DD_14.17_ in all sites except OR, which coincides with the thermal requirements it takes *H. halys* to progress from egg to reproductive maturity (606–681 DD_14.17_). This increase extended into earlier time frames (beginning at 450 DD_14.17_) in Oregon which may be a sampling artifact or related to genetic differences in the West coast populations or environmental factors. Due to the milder climate in Oregon, it is possible that *H. halys* does not undergo facultative diapause, which would allow reproduction earlier in the spring and may explain the rather flat rate of vitellogenesis observed.

Analysis of field‐collected specimens identified that the seasonal phenology of adult females—the rate at which they progressed to vitellogenic status—is indeed different between geographic locations, at least within the overwintering generation (up to 650 DD_14.17_) where diapause termination and early season population growth commences. This may reflect phenotypic patterns acted on by natural selection during spread of this invasive species (Bradshaw & Holzapfel, 2001; Urbanski et al., 2012). Specifically, environmental factors such as host availability or nutrition during spring population growth and dispersal. Geographic differences in phenotypic patterns has occurred rapidly in invasive species or in response to climate change (Bradshaw & Holzapfel, 2001; Colautti & Barrett, 2013). Phenotypic differences are evident in the US population of *H. halys*, which is believed to have been in the US for at least 20 years. We accounted for differences in temperature and photoperiod through our model but factors such as differential availability of host plants as adults leave overwintering sites, sampling effort, population genetics, or even differences in critical diapause cues may be responsible for this variation. Further work is needed to determine what processes are driving the variation in the rate at which the population transitions into a vitellogenic status, and the methods presented here may facilitate those studies.

The differences in phenotypic patterns observed in this study were predicted by the phenological model by Nielsen et al. (2016). This model predicted significantly different population sizes, and proportions of *F*_1_ and *F*_2_ adults entering overwintering, across eight geographic locations within the US. Although initially we hoped our field results presented here would be able to clearly separate generations and validate the predicted bivoltinism, due to the long lifespan of *H. halys* and continuous reproduction, we were unable to do so, despite recording the corpus luteum. Our field results did however confirm a strong overlap of adults among generations, which is consistent with modeled results (Nielsen et al., 2016). The stage‐specific model, however, utilized a 13.5‐hr day length to terminate diapause, and our results suggest this should be shortened to 12.7 hr, which would increase the duration of the year in which the population would be reproductively active. Our results also provide evidence that reproductive females do not revert to a previtellogenic state and subsequently diapause, supporting assumptions made by Nielsen et al. (2016) in the stage‐specific phenology model. Field collections from light traps or pheromone traps of other pentatomid species also suggest that females primarily overwinter in a nonreproductive state (Cullen & Zalom, 2006; Katayama et al., 1993; Toscano & Stern, 1980). Adoption of such a life history strategy may help optimize reproductive fitness and overwintering survival. Although oosorption has been demonstrated by young *Plautia stali* in the laboratory (Kotaki, Kaihara, Ando, Misaki, & Shinada, 2016), there is no evidence for oosorption in older females entering diapause.

Our results indicate that some aspects of *H. halys* phenology are conserved across regions. Specifically, we saw emergence from and entrance into overwintering habitats in a previtellogenic state; a sequential development of reproductive ranks, and increasing presence of previously mated females. This provides strong evidence that females are entering into facultative diapause in the locations evaluated and that, as in other Pentatomidae, reproductive diapause is triggered by critical photoperiod (Musolin & Saulich, 2012). Given the current research efforts focused on *H. halys* due to its global pest status, it would make a good model species to understand reproductive physiology and diapause cues in the Pentatomidae.

## CONFLICT OF INTEREST

None declared.

## AUTHOR CONTRIBUTIONS

ALN designed experiments, collected data, developed classification of reproductive development, analyzed dataset; JMP refined the *SD*_DD_ method and developed R code; SJF and TCL assisted with interpretation of data for the work; All authors collected specimens and acquisition of data and provided editorial comments on manuscript draft. All authors are accountable for all aspects of the work.

## References

[ece33125-bib-0001] Acebes‐Doria, A. L. , Leskey, T. C. , & Bergh, J. C. (2016). Host plant effects on *Halyomorpha halys* (Hemiptera: Pentatomidae) nymphal development and survivorship. Environmental Entomology, 45, 663–670. 10.1093/ee/nvw018 27012749

[ece33125-bib-0002] Association, U. U. S. A. (2011). Losses to mid‐Atlantic apple growers at $37 million from brown marmorated stink bug. Vienna, VA: United States Apple Association.

[ece33125-bib-0003] Baskerville, G. L. , & Emin, P. (1969). Rapid estimation of heat accumulation from maximum and minimum temperatures. Ecology, 50, 514–516.

[ece33125-bib-0004] Bradshaw, W. E. , & Holzapfel, C. M. (2001). Genetic shift in photoperiodic response correlated with global warming. Proceedings of the National Academy of Sciences, 98, 14509–14511. 10.1073/pnas.241391498 PMC64712 11698659

[ece33125-bib-0005] Colautti, R. I. , & Barrett, S. C. H. (2013). Rapid adaptation to climate facilitates range expansion of an invasive plant. Science, 342, 364–366. 24136968 10.1126/science.1242121

[ece33125-bib-0006] Cullen, E. M. , & Zalom, F. G. (2006). *Euschistus conspersus* female morphology and attraction to methyl (2*E*,4*Z*)‐decadienoate pheromone‐baited traps in processing tomatoes. Entomologia Experimentalis et Applicata, 119, 163–173.

[ece33125-bib-0007] Danks, H. V. (2007). The elements of seasonal adaptations in insects. The Canadian Entomologist, 139, 1–44.

[ece33125-bib-0008] Esquivel, J. F. (2009). Stages of gonadal development of the southern green stink bug (Hemiptera: Pentatomidae): Improved visualization. Annals of the Entomological Society of America, 102, 303–309.

[ece33125-bib-0009] Gariepy, T. D. , Fraser, H. , & Scott‐Dupree, C. D. (2014). Brown marmorated stink bug (Hemiptera: Pentatomidae) in Canada: Recent establishment, occurrence, and pest status in southern Ontario. The Canadian Entomologist, 146, 579–582.

[ece33125-bib-0010] Haye, T. , Abdallah, S. , Gariepy, T. , & Wyniger, D. (2014). Phenology, life table analysis and temperature requirements of the invasive brown marmorated stink bug, *Halyomorpha halys*, in Europe. Journal of Pest Science, 87, 407–418.

[ece33125-bib-0011] Hoebeke, E. R. , & Carter, M. E. (2003). *Halyomorpha halys* (Stal) (Heteroptera: Pentatomidae): A polyphagous plant pest from Asia newly detected in North America. Proceedings of the Entomological Society of Washington, 105, 225–237.

[ece33125-bib-0012] Katayama, E. , Fukuda, T. , & Nozawa, H. (1993). Light trap monitoring of the fruit tree stink bugs and their ovarian development. Tochigi Agricultural Research Report, 59–74.

[ece33125-bib-0013] Kiritani, K. (1963). The change in reproductive system of the southern green stink bug, Nezara viridula, and its application to forecasting of the seasonal history. Japanese Journal of Applied Entomology and Zoology, 7, 327–337.

[ece33125-bib-0014] Koštál, V. (2011). Insect photoperiodic calendar and circadian clock: Independence, cooperation, or unity? Journal of Insect Physiology, 57, 538–556. http://www.sciencedirect.com/science/article/pii/S0022191010002970 21029738 10.1016/j.jinsphys.2010.10.006

[ece33125-bib-0015] Kotaki, T. , Kaihara, K. , Ando, Y. , Misaki, K. , & Shinada, T. (2016). Oosorption in the stink bug Plautia stali: Role of Juvenile hormone in the induction of oosorption. Physiological Entomology, 41, 127–141.

[ece33125-bib-0016] Lee, C. E. (2002). Evolutionary genetics of invasive species. Trends in Ecology and Evolution, 17, 386–391.

[ece33125-bib-0017] Leskey, T. C. , Hamilton, G. C. , Nielsen, A. L. , Polk, D. F. , Rodriguez‐Saona, C. , Bergh, J. C. , … Wright, S. E. (2012). Pest status of the Brown marmorated stink bug, *Halyomorpha halys* in the USA. Outlooks on Pest Management, 23, 218–226.

[ece33125-bib-0018] Leskey, T. C. , Short, B. D. , Butler, B. R. , & Wright, S. E. (2012). Impact of the invasive brown marmorated stink bug, *Halyomorpha halys* (Stål), in Mid‐Atlantic tree fruit orchards in the United States: Case studies of commercial management. Psyche: A Journal of Entomology, 2012, 1–14.

[ece33125-bib-0019] Maistrello, L. , Dioli, P. , Bariselli, M. , Mazzoli, G. L. , & Giacalone‐Forini, I. (2016). Citizen science and early detection of invasive species: Phenology of first occurrences of *Halyomorpha halys* in Southern Europe. Biological Invasions, 1–8.

[ece33125-bib-0020] Musolin, D. , & Saulich, A. (2012). Responses of insects to the current climate changes: From physiology and behavior to range shifts. Entomological Review, 92, 715–740. http://www.springerlink.com/content/yn8q2px747g757r4/abstract/

[ece33125-bib-0021] Nielsen, A. L. , Fleischer, S. , & Chen, S. (2016). Coupling developmental physiology, photoperiod, and temperature to model phenology and dynamics of an invasive Heteropteran, *Halyomorpha halys* . Frontiers in Physiology, 7, 165. 27242539 10.3389/fphys.2016.00165 PMC4870838

[ece33125-bib-0022] Nielsen, A. L. , & Hamilton, G. C. (2009a). Life history of the invasive species *Halyomorpha halys* (Hemiptera: Pentatomidae) in Northeastern United States. Annals of the Entomological Society of America, 102, 608–616.

[ece33125-bib-0023] Nielsen, A. L. , & Hamilton, G. C. (2009b). Seasonal occurrence and impact of *Halyomorpha halys* (Hemiptera: Pentatomidae) in tree fruit. Journal of Economic Entomology, 102, 1133–1140. 19610429 10.1603/029.102.0335

[ece33125-bib-0024] Nielsen, A. L. , Hamilton, G. C. , & Matadha, D. (2008). Developmental rate estimation and life table analysis for *Halyomorpha halys* (Hemiptera: Pentatomidae). Environmental Entomology, 27, 348–355. 10.1603/0046-225x(2008)37[348:drealt]2.0.co;2 18419906

[ece33125-bib-0025] Niva, C. C. , & Takeda, M. (2003). Effects of photoperiod, temperature and melatonin on nymphal development, polyphenism and reproduction in *Halyomorpha halys* (Heteroptera: Pentatomidae). Zoological Science, 20, 963–970. 12951401 10.2108/zsj.20.963

[ece33125-bib-0026] Numata, H. (2004). Environmental factors that determine the seasonal onset and termination of reproduction in seed‐sucking bugs (Heteroptera) in Japan. Applied Entomolgical Zoology, 39, 565–573.

[ece33125-bib-0027] Ogburn, E. C. , Bessin, R. , Dieckhoff, C. , Dobson, R. , Grieshop, M. , Hoelmer, K. A. , … Walgenbach, J. F. (2016). Natural enemy impact on eggs of the invasive brown marmorated stink bug, *Halyomorpha halys* (Stål) (Hemiptera: Pentatomidae), in organic agroecosystems: A regional assessment. Biological Control, 101, 39–51.

[ece33125-bib-0028] Pote, J. M. , Nielsen, A. L. , & Grieshop, M. J. (2016). Biology and seasonality of the reemergent pest *Rhynchaenus pallicornis* (Coleoptera: Curculionidae) and methods for monitoring its abundanc. Enviromental Entomology, 1–9. 10.1093/ee/nvw029 27288674

[ece33125-bib-0029] Rice, K. B. , Bergh, C. J. , Bergmann, E. J. , Biddinger, D. J. , Dieckhoff, C. , Dively, G. , … Tooker, J. F. (2014). Biology, ecology, and management of brown marmorated stink bug (Hemiptera: Pentatomidae). Journal of Integrated Pest Management, 5, A1–A13.

[ece33125-bib-0030] SASInstitute (2008). JMP 8.0. Cary, NC.

[ece33125-bib-0032] Snyder, R. L. , Spano, D. , Cesaraccio, C. , & Duce, P. (1999). Determining degree‐day thresholds from field observations. International Journal of Biometeorology, 42, 177–182. 10.1007/s004840100104 11769315

[ece33125-bib-0033] Tauber, M. J. , & Tauber, C. A. (1976). Insect seasonality: Diapause maintenance, termination, and postdiapause development. Annual Review of Entomology, 21, 81–107. https://doi.org/www.annualreviews.org/doi/abs/10.1146/annurev.en.21.010176.000501

[ece33125-bib-0034] Toscano, N. C. , & Stern, V. M. (1980). Seasonal reproductive condition of *Euschistus conspersus* . Annals of the Entomological Society of America, 73, 85–88.

[ece33125-bib-0035] Urbanski, J. , Mogi, M. , O'Donnell, D. , Decotiis, M. , Toma, T. , & Armbruster, P. (2012). Rapid adaptive evolution of photoperiodic response during invasion and range expansion across a climatic gradient. The American Naturalist, 179, 490–500. 10.1086/664709 22437178

[ece33125-bib-0036] Valentin, R. E. , Nielsen, A. L. , Wiman, N. G. , Lee, D.‐H. , & Fonseca, D. M. (in revision). Global invasion network: Identifying invasion pathways of the brown marmorated stink bug. Halyomorpha halys (Stål). 10.1038/s41598-017-10315-z PMC5575200 28852110

[ece33125-bib-0037] Watanabe, M. (1979). Ecology and extermination of *Halyomorpha halys*. 4. The relationship between day length and ovarian development. Annual Report of Toyama Institute of Health, 3, 33–27.

[ece33125-bib-0038] Wolda, H. (1988). Insect seasonality: Why? Annual Review of Ecology and Systematics, 19, 1–18.

[ece33125-bib-0039] Xu, J. , Fonseca, D. M. , Hamilton, G. C. , Hoelmer, K. A. , & Nielsen, A. L. (2014). Tracing the origin of US brown marmorated stink bugs, *Halyomorpha halys* . Biological Invasions, 16, 153–166.

[ece33125-bib-0040] Yanagi, T. , & Hagihara, Y. (1980). Ecology of the brown marmorated stink bug. Plant Protection, 34, 315–326.

[ece33125-bib-0041] Zheng, D. B. , Hu, G. , Yang, F. , Du, X. D. , Yang, H. B. , Zhang, G. , … Zhai, B. P. (2014). Ovarian development status and population characteristics of *Sogatella furcifera* (Horváth) and *Nilaparvata lugens* (Stål): Implications for pest forecasting. Journal of Applied Entomology, 138, 67–77.

