# Supplementary material for: Phenology of brown marmorated stink bug described using female reproductive development
Source: Ecol Evol. 2017 Jul 21;7(17):6680–90. doi: 10.1002/ece3.3125 (PMC5587477; doi:10.1002/ece3.3125)
Supplement: Supplementary file 1 [file ECE3-7-6680-s001.docx]

**Supplemental Table 1**. A summary of the calculation of the sample size scalar, $p_{i}$, used to determine the modified standard deviation in degree-days (SD_DD_). (NC= Asheville, NC; NJ= Bridgeton, NJ, OR= Willamette Valley, OR; PAA= Allentown, PA, PAB= Biglerville, PA; WV= Kearneysville, WV).

| **Location** | **Year** | **Females collected up to and including date of critical vitellogenesis** | **Proportion of pre-critical vitellogenesis sampling (*p_i_*)** |
| --- | --- | --- | --- |
| NC | 2013 | 6 | 0.0050 |
| NC | 2014 | 47 | 0.0392 |
| NJ | 2012 | 98 | 0.0817 |
| NJ | 2013 | 231 | 0.1927 |
| NJ | 2014 | 169 | 0.1410 |
| OR | 2012 | 10 | 0.0083 |
| OR | 2013 | 1 | 0.0008 |
| OR | 2014 | 21 | 0.0175 |
| PAA | 2006 | 6 | 0.0050 |
| PAA | 2007 | 3 | 0.0025 |
| PAA | 2008 | 7 | 0.0058 |
| PAA | 2012 | 204 | 0.1701 |
| PAA | 2013 | 198 | 0.1651 |
| PAB | 2013 | 67 | 0.0559 |
| WV | 2012 | 44 | 0.0367 |
| WV | 2013 | 68 | 0.0567 |
| WV | 2014 | 19 | 0.0158 |
| **Total** | | 1199 |  |

**Supplemental Table 2.** Results of the modified Snyder model to identify biofix for *H. halys* females reaching 10% vitellogenesis. The lowest standard deviation value selected as the biofix is 12.7 h photoperiod is highlighted in grey.

| Diapause Termination Cue | Modified Standard Deviation of Model Outputs |
| --- | --- |
| **1-Jan** | 12.006 |
| **12.0** | 11.217 |
| **12.1** | 10.861 |
| **12.2** | 10.573 |
| **12.3** | 10.322 |
| **12.4** | 10.311 |
| **12.5** | 10.323 |
| **12.6** | 10.305 |
| **12.7** | 10.285 |
| **12.8** | 10.353 |
| **12.9** | 10.298 |
| **13.0** | 11.023 |
| **13.1** | 10.662 |
| **13.2** | 10.555 |
| **13.3** | 10.811 |
| **13.4** | 10.681 |
| **13.5** | 10.547 |
| **13.6** | 10.416 |
| **13.7** | 10.445 |
| **13.8** | 10.482 |
| **13.9** | 10.610 |
| **14.0** | 10.768 |
| **14.1** | 668.422 |
| **14.2** | 52806.496 |
| **14.3** | 4841108.210 |
| **14.4** | 507150600.1000 |
| **14.5** | 54175462617.000 |

**Supplemental Table 3.** Total number of females collected and assigned to a reproductive rank during the sampling period. (NC= Asheville, NC; NJ= Bridgeton, NJ, OR= Willamette Valley, OR; PAA= Allentown, PA, PAB= Biglerville, PA; WV= Kearneysville, WV).

|  | **Location** | | | | | |
| --- | --- | --- | --- | --- | --- | --- |
| **Accumulated DD_14_** | **NC** | **NJ** | **OR** | **PAA** | **PAB** | **WV** |
| 0 | 0 | 75 | 0 | 0 | 0 | 0 |
| 50 | 5 | 116 | 24 | 120 | 0 | 39 |
| 100 | 6 | 177 | 23 | 167 | 0 | 51 |
| 150 | 23 | 97 | 24 | 48 | 35 | 31 |
| 200 | 19 | 68 | 1 | 133 | 59 | 27 |
| 250 | 30 | 61 | 9 | 71 | 60 | 33 |
| 300 | 10 | 60 | 12 | 61 | 20 | 23 |
| 350 | 23 | 27 | 26 | 55 | 89 | 35 |
| 400 | 7 | 28 | 7 | 37 | 26 | 5 |
| 450 | 16 | 15 | 8 | 41 | 0 | 7 |
| 500 | 10 | 7 | 8 | 25 | 5 | 0 |
| 550 | 11 | 17 | 5 | 20 | 0 | 0 |
| 600 | 12 | 10 | 0 | 31 | 0 | 18 |
| 650 | 43 | 2 | 7 | 59 | 3 | 19 |
| 700 | 36 | 42 | 12 | 38 | 2 | 2 |
| 750 | 86 | 30 | 20 | 85 | 17 | 29 |
| 800 | 56 | 46 | 0 | 117 | 0 | 16 |
| 850 | 43 | 26 | 0 | 101 | 19 | 7 |
| 900 | 45 | 74 | 0 | 168 | 37 | 22 |
| 950 | 20 | 63 | 0 | 123 | 16 | 55 |
| 1000 | 39 | 60 | 0 | 175 | 50 | 18 |
| 1050 | 14 | 101 | 0 | 158 | 16 | 62 |
| 1100 | 0 | 76 | 0 | 155 | 0 | 10 |
| 1150 | 0 | 94 | 0 | 37 | 85 | 11 |
| 1200 | 0 | 117 | 0 | 20 | 4 | 32 |
| 1250 | 0 | 123 | 0 | 0 | 0 | 0 |
| 1300 | 0 | 1 | 0 | 0 | 0 | 0 |
| 1350 | 0 | 15 | 0 | 0 | 0 | 0 |
